# Supplementary material for: IA-Body Composition CT at T12 in Idiopathic Pulmonary Fibrosis: Diagnosing Sarcopenia and Correlating with Other Morphofunctional Assessment Techniques
Source: Nutrients. 2024 Aug 28;16(17):2885. doi: 10.3390/nu16172885 (PMC11396836; doi:10.3390/nu16172885)
Supplement: Supplementary file 1 [file nutrients-16-02885-s001.zip › nutrients-3158828-supplementary.pdf]

Figure S1. Sample selection of patients with pulmonary fibrosis for study.

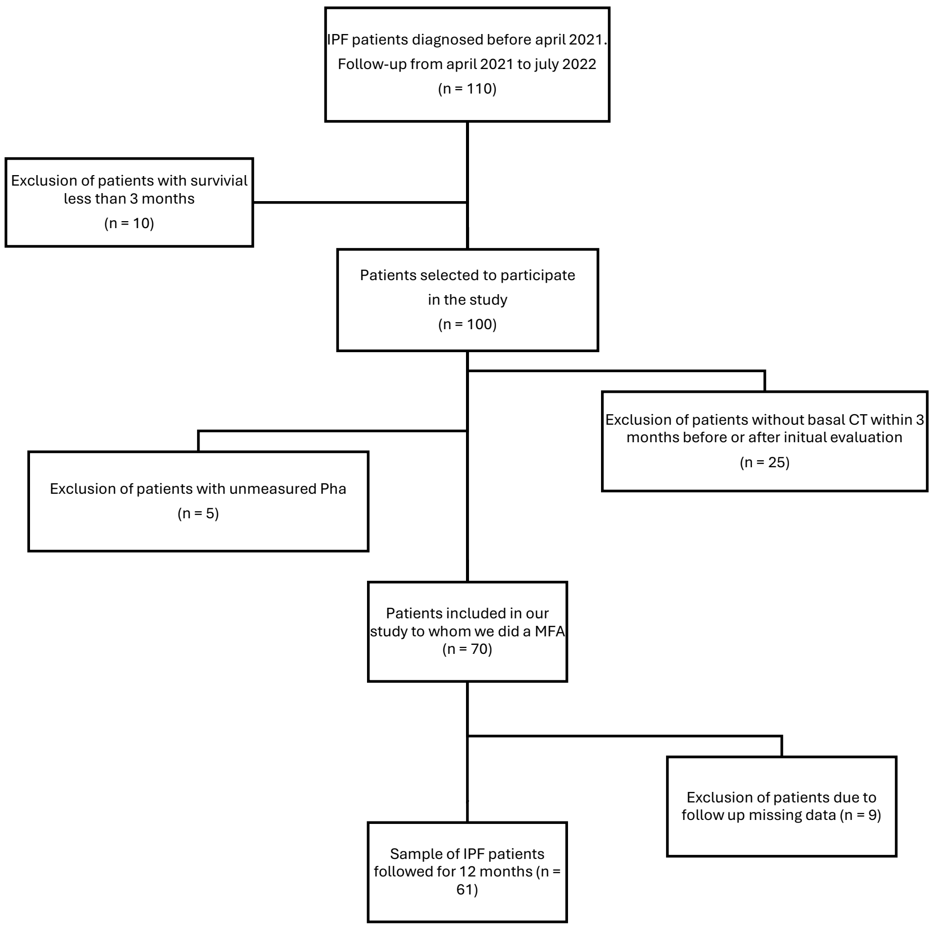

Abbreviations: CT (computed tomography), MFA (morphofunctional assessment); Pha (phase angle); IPF (idiopathic pulmonary fibrosis).
